# Elevated Inflammatory Burden Index Is Association With Increased Sarcopenia: A Population‐Based Study

**DOI:** 10.1155/mi/9991220

**Published:** 2026-03-26

**Authors:** Li Xiao, Yunzhe Li, Xiuyun Li, Zixin Luo, Chenxi Wang, Shaochun Liu, Nan Huang, Maoyuan Wang, Kang Zou

**Affiliations:** ^1^ Department of Rehabilitation Medicine, The First Affiliated Hospital of Gannan Medical University, Ganzhou City, Jiangxi Province, 341000, People′s Republic of China, gmu.cn; ^2^ School of Rehabilitation Medicine, Gannan Medical University, Ganzhou City, Jiangxi Province, 341000, People′s Republic of China, gmu.cn; ^3^ Ganzhou Key Laboratory of Rehabilitation Medicine, Gannan Medical University, Ganzhou City, Jiangxi Province, 341000, People′s Republic of China, gmu.cn; ^4^ Ganzhou Intelligent Rehabilitation Technology Innovation Center, Gannan Medical University, Ganzhou City, Jiangxi Province, 341000, People′s Republic of China, gmu.cn; ^5^ The Second Clinical Medical College of Zhejiang Chinese Medical University, Hangzhou City, Zhejiang Province, 310000, People′s Republic of China, zcmu.edu.cn; ^6^ The First Clinical Medical College, Gannan Medical University, Ganzhou City, Jiangxi Province, 341000, People′s Republic of China, gmu.cn; ^7^ Department of Critical Care Medicine, The First Affiliated Hospital of Gannan Medical University, Ganzhou City, Jiangxi Province, 341000, People′s Republic of China, gmu.cn

**Keywords:** C-reactive protein, inflammatory burden index, neutrophil-to-lymphocyte ratio, NHANES, sarcopenia

## Abstract

**Background:**

The inflammatory burden index (IBI) is a comprehensive indicator of the inflammatory state of the body and is associated with a variety of chronic diseases. Sarcopenia is a disease characterized by a reduction in skeletal muscle mass, but the association between IBI and sarcopenia is currently unclear.

**Methods:**

This study was based on data from the National Health and Nutrition Examination Survey (NHANES) 2015–2018 and included 4523 participants aged 20 years and older. IBI was calculated by the product of C‐reactive protein (CRP) and neutrophil‐to‐lymphocyte ratio (NLR). Sarcopenia was defined by the extremity skeletal muscle mass index (ASM/body mass index [BMI]). The association between IBI and sarcopenia was analyzed using multivariate logistic regression models with nonlinear and subgroup analyses.

**Results:**

The mean age of the participants was 39.9 years, and 52.5% were female. Higher IBI scores were associated with a higher risk of chronic disease. IBI was positively associated with sarcopenia, with the highest IBI group having a 1.94 times greater risk of sarcopenia than the lowest group (95% CI: 1.34–2.81). The natural log transformation of IBI resulted in a 42% increase in risk of sarcopenia for each unit increase (95% CI: 1.08–1.87). Nonlinear analyses showed an inflection point in the association between IBI and sarcopenia at 2.38, with a significant increase in risk before the inflection point and no longer significant after the inflection point. Subgroup analyses showed that this association was consistent across sex, age, diabetes, cardiovascular disease (CVD), and chronic kidney disease (CKD).

**Conclusion:**

There is a positive association between IBI and sarcopenia with nonlinear characteristics. High IBI levels may increase the risk of sarcopenia, suggesting that inflammation may play an important role in sarcopenia, providing a potential target for future interventions.

## 1. Introduction

Sarcopenia is a syndrome characterized by the loss of skeletal muscle mass, strength, and function due to aging or other factors, which often affects the quality of life and physical function in older adults [1, 2]. A considerable corpus of epidemiological data suggests that ~10%–30% of the global elderly population is affected by this disease [3]. It is also noteworthy that the middle‐aged population is at increased risk for sarcopenia [4, 5]. With the acceleration of global aging, sarcopenia has attracted widespread attention and has become an important research topic in public health.

The Inflammatory Burden Index (IBI) is a powerful tool for quantifying the inflammatory status of individuals and populations. It is designed to assess the degree of chronic inflammation in the body. The IBI takes into account a variety of inflammatory markers (e.g., levels of serum inflammatory factors, acute phase reactive proteins, immune cell types, etc.) and provides a new way of investigating the path mechanisms of complex diseases [6, 7]. Chronic inflammation is considered to be an important contributing factor to many common diseases in the elderly (e.g., cardiovascular disease (CVD), diabetes, obesity, etc.). It exerts a profound influence on the metabolic and functional status of the body [8–10].

In this context, mounting research has identified a potential association with sarcopenia and inflammation burden [11, 12]. Chronic low‐grade inflammation may lead to further loss of muscle mass and strength through a variety of mechanisms, such as decreasing muscle synthesis, promoting muscle degradation, etc. [13]. Traditional indicators of inflammation such as the systemic inflammatory index (SII), white blood cell counts, C‐reactive protein (CRP), cytokine levels, etc., while providing important information for assessing the state of inflammation in vivo, often appear to be incomplete [14, 15]. These traditional metrics tend to focus on the overall state of inflammation, and it is difficult to reflect the complex roles of inflammation in muscle metabolism. Conversely, the IBI, as a more comprehensive quantitative tool, integrates multiple inflammatory markers, thereby facilitating meticulous assessment of the impact of chronic inflammation in vivo. While traditional inflammatory markers may be more suitable for broad pathological state monitoring, the IBI is more advantageous in studies targeting mechanisms of action in muscle health.

In summary, an exploration of the association between sarcopenia and the IBI is of significant value in enhancing our comprehension of the mechanisms underlying this complex disease, as well as in delineating novel research directions for preventing and treating sarcopenia. The exploration of this connection can facilitate the development of novel intervention strategies aimed at reducing the incidence of sarcopenia and enhancing the quality of life of affected individuals.

## 2. Materials and Methods

### 2.1. Study Participant Data Sources and Presentations

**Figure 1:**
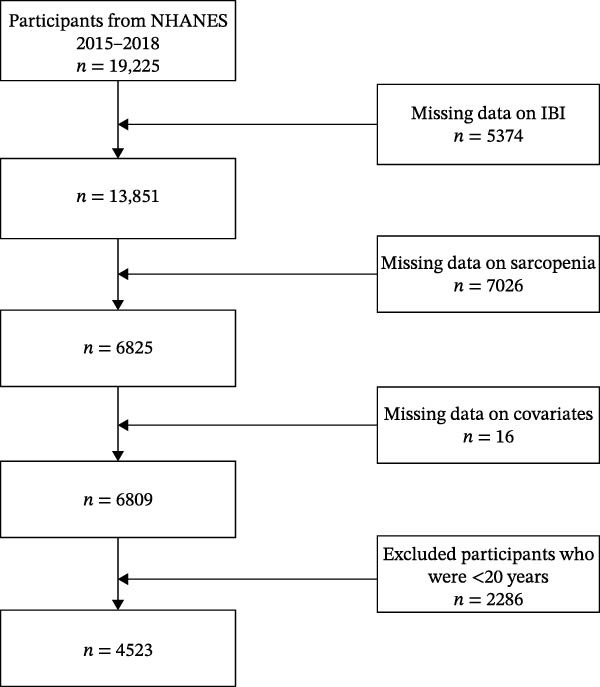
Selection of study participants.

The National Health and Nutrition Examination Survey (NHANES) represents a large‐scale national study that assesses the health and nutritional status of Americans through the use of questionnaires, physical examinations, and laboratory tests. The present study incorporated a total of 19,225 participants from both cycles from 2015 to 2018. Initial exclusion criteria included participants lacking IBI‐related data or exhibiting abnormalities (*n* = 5392). Thereafter, individuals with abnormal and missing data related to sarcopenia were excluded (*n* = 7026), as were participants with missing covariates (*n* = 1233). Finally, participants who have not yet reached the age of 20 were also excluded (*n* = 1051). The final sample consisted of 4523 individuals (Figure 1). It is worth noting that all protocols were approved by the National Center for Health Statistics (NCHS) Research Ethics Review Board, and this study was exempt from ethical approval. All individuals participating in the NHANES survey provided written informed consent.

In this study, the laboratory procedures defined by NHANES were strictly followed. The neutrophil‐to‐lymphocyte ratio (NLR) was calculated for each participant by dividing the absolute neutrophil count by the absolute lymphocyte count. We used a Behring nephelometer to quantify the levels of CRP. IBI = CRP × neutrophils/lymphocytes.

### 2.2. Definition of Sarcopenia

We used data from the physical examination portion of NHANES to extract values for limb skeletal muscle mass (ASM), which represents the total lean mass of the limbs. The muscle loss index (ASM/body mass index [BMI]) was not applicable to pregnant women, individuals who were too tall (>192.5 cm) or too heavy (>136.4 kg). Sarcopenia is defined when the ASM/BMI is less than 0.512 for women and less than 0.789 for men.

### 2.3. Covariates Acquisition

Covariates included demographic data such as participants’ sex, age, race, education level, and poverty‐income ratio; anthropometric data such as BMI; and laboratory data including high‐density lipoprotein cholesterol (HDL‐C), low‐density lipoprotein cholesterol (LDL‐C), neutrophil count, lymphocyte count, total serum calcium, and total cholesterol. Alcohol intake and smoking were both categorized into three categories according to smoking and drinking status in the participants’ NHANES questionnaire section. Current drinking (12 or more drinks in a year), ever drinking (less than 12 drinks in a year, but at least 12 drinks in their lifetime), and never drinking (less than 12 drinks in a lifetime). Current smoker (now smokes daily as well as sometimes), ever smoker (smoked at least 12 times in their lifetime but is currently a nonsmoker), and never smoker (smoked less than 12 times in total and is currently a nonsmoker). Hypertension was judged based on the following criteria: (1) self‐reported history of hypertension diagnosed by a doctor; (2) systolic blood pressure ≥140 mmHg; (3) diastolic blood pressure ≥90 mmHg; and (4) taking antihypertensive drugs. Diabetes mellitus was determined by: (1) self‐reported history of diabetes diagnosed by a doctor; (2) fasting blood glucose level of 126 mg/dL or higher; (3) 2‐h oral glucose tolerance test showing glucose levels at or above 200 mg/dL; (4) HbA1c ≥6.5%; and (5) the use of insulin or antidiabetic drugs. CVD was judged on the basis of whether you had ever suffered from congestive heart failure, angina pectoris, myocardial infarction, coronary artery disease, or stroke, and those who experienced any of these conditions were classified as having CVD. Chronic kidney disease (CKD) was judged by (1) eGFR <60 mL/min/1.73 m^2^ and (2) UACR ≥30 mg/g. Covariates were selected on the basis that they made the effect value of *x* affect more than 10%, as well as a one‐way regression *p*‐value of <0.50 (Table S1), and ultimately the decision to include them in the analysis was made in the context of previous studies and clinical significance.

### 2.4. Statistical Analysis

We performed statistical analysis of the data following the guidelines from the CDC. Weights were chosen as 2‐year MEC weights for the fasting subsample and divided by the number of cycles to account for oversampling of minorities, thus ultimately providing unbiased and accurate effect estimates. We categorized IBI into four groups (<4.29, 4.29–6.85, 6.85–12.34, and >12.34) based on previous studies describing the baseline characteristics of the enrolled population. Continuous variables are presented as mean ± standard deviation, while categorical variables are expressed as percentages. Comparisons between groups are conducted by employing the *t*‐test or one‐way analysis of variance. Furthermore, a range of comparative analyses between designated groups is undertaken by the SNK or LSD methodologies. We used multivariate binary logistic regression models to derive OR and 95% CI and performed ln transformations for IBI, as well as quadratic groupings for analyses. Three different models were used to evaluate the link between IBI levels and sarcopenia. Crude model was unadjusted. Model 1 was subjected to adjustment for sex, age, race, education, and PIR. Model 2 was subjected to adjustment for BMI, total serum calcium, HDL, LDL, total cholesterol, alcohol intake, smoking status, hypertension, diabetes mellitus, CVD, and CKD based on Model 1. Also, for the stability of the results, we performed sensitivity analyses and included the missing covariates in the regression again after multiple interpolations (at least five times). To explore the nonlinear association between IBI and sarcopenia, complex weighted RCS curves were used, and inflection points were calculated. Finally, a series of classified analyses were conducted to ascertain the impact of variables in various subgroups in association with the association between IBI and sarcopenia. All analyses were conducted using R Version 3.3.2 and Free Statistics Version 2.0.

## 3. Result

### 3.1. Baseline Features of the Population

The IBI is described in four groups (<4.29, 4.29–6.85, 6.85–12.34, and >12.34). Participants were slightly more female (52.5%). The participants’ average age was 39.9, and non‐Hispanic whites made up the largest proportion. Participants in the highest IBI subgroups had a higher risk of chronic diseases such as CVD compared to the other groups (Table 1).

**Table 1:** Baseline characteristics of participants.

Variables	Inflammatory burden index				
	Total (*n* = 4523)	<4.29 (*n* = 1128)	4.29–6.85 (*n* = 1131)	6.85–12.34 (*n* = 1131)	>12.34 (*n* = 1133)
Sex, *n* (%)					
Male	2147 (47.5)	860 (76.2)	562 (49.7)	381 (33.7)	344 (30.4)
Female	2376 (52.5)	268 (23.8)	569 (50.3)	750 (66.3)	789 (69.6)
Age (year)	39.9 ± 11.5	37.9 ± 11.0	39.9 ± 11.6	40.0 ± 11.5	41.7 ± 11.5
Race, *n* (%)					
Mexican American	809 (17.9)	196 (17.4)	211 (18.7)	208 (18.4)	194 (17.1)
Other Hispanic	551 (12.2)	146 (12.9)	126 (11.1)	143 (12.6)	136 (12)
Non‐Hispanic White	1358 (30.0)	331 (29.3)	362 (32)	345 (30.5)	320 (28.2)
Non‐Hispanic Black	888 (19.6)	211 (18.7)	218 (19.3)	186 (16.4)	273 (24.1)
Other race	917 (20.3)	244 (21.6)	214 (18.9)	249 (22)	210 (18.5)
Education level, *n* (%)					
Nongraduate	1865 (41.2)	450 (39.9)	458 (40.5)	453 (40.1)	504 (44.5)
High school graduation	2658 (58.8)	678 (60.1)	673 (59.5)	678 (59.9)	629 (55.5)
PIR, *n* (%)					
≤1	929 (20.5)	186 (16.5)	230 (20.3)	246 (21.8)	267 (23.6)
>1	3594 (79.5)	942 (83.5)	901 (79.7)	885 (78.2)	866 (76.4)
BMI, mean ± SD	29.1 ± 6.5	29.9 ± 5.7	29.0 ± 6.0	28.5 ± 6.6	29.1 ± 7.3
Total calcium, mean ± SD	9.3 ± 0.3	9.3 ± 0.3	9.3 ± 0.3	9.3 ± 0.3	9.3 ± 0.4
HDL‐C, mean ± SD	52.3 ± 15.6	47.9 ± 13.6	52.3 ± 14.8	54.4 ± 15.4	54.6 ± 17.4
LDL‐C, mean ± SD	102.5 ± 38.1	105.6 ± 39.0	103.5 ± 37.1	99.0 ± 38.0	101.8 ± 37.8
Total cholesterol, mean ± SD	190.7 ± 39.6	193.0 ± 40.2	189.9 ± 38.8	189.4 ± 39.3	190.6 ± 39.9
Alcohol intake, *n* (%)					
Current	2021 (44.7)	529 (46.9)	533 (47.1)	493 (43.6)	466 (41.1)
Former	1933 (42.7)	485 (43)	472 (41.7)	479 (42.4)	497 (43.9)
Never	569 (12.6)	114 (10.1)	126 (11.1)	159 (14.1)	170 (15)
Smoking status, *n* (%)					
Current	2372 (52.4)	565 (50.1)	585 (51.7)	615 (54.4)	607 (53.6)
Former	747 (16.5)	201 (17.8)	209 (18.5)	165 (14.6)	172 (15.2)
Never	1404 (31.0)	362 (32.1)	337 (29.8)	351 (31)	354 (31.2)
Hypertension, *n* (%)					
No	1054 (23.3)	209 (18.5)	230 (20.3)	253 (22.4)	362 (32)
Yes	3469 (76.7)	919 (81.5)	901 (79.7)	878 (77.6)	771 (68)
Diabetes, *n* (%)					
No	4175 (92.3)	1082 (95.9)	1064 (94.1)	1054 (93.2)	975 (86.1)
Yes	348 (7.7)	46 (4.1)	67 (5.9)	77 (6.8)	158 (13.9)
CVD, *n* (%)					
No	4347 (96.1)	1093 (96.9)	1096 (96.9)	1094 (96.7)	1064 (93.9)
Yes	176 (3.9)	35 (3.1)	35 (3.1)	37 (3.3)	69 (6.1)
CKD, *n* (%)					
No	4117 (91.0)	1119 (99.2)	1124 (99.4)	1121 (99.1)	753 (66.5)
Yes	406 (9.0)	9 (0.8)	7 (0.6)	10 (0.9)	380 (33.5)
Sarcopenia, *n* (%)					
No	4059 (89.7)	1034 (91.7)	1020 (90.2)	1017 (89.9)	988 (87.2)
Yes	464 (10.3)	94 (8.3)	111 (9.8)	114 (10.1)	145 (12.8)

*Note:* Survey data were presented as weighted means ± standard errors for continuous variables, and unweighted frequencies (weighted proportions) for categorical variables.

Abbreviations: BMI, body mass index; CKD, chronic kidney disease; CVD, cardiovascular disease; HDL‐C, high‐density lipoprotein‐cholesterol; IBI, inflammatory burden index; LDL‐C, low‐density lipoprotein‐cholesterol; PIR, poverty‐to‐income ratio.

### 3.2. Association Between IBI and Sarcopenia

The positive association concerning IBI and sarcopenia was found to be unchanged when IBI was treated differently. When IBI was quadrupled, the risk of developing sarcopenia (OR: 1.94, 95% CI: 1.34–2.81) was much higher in the highest group than in the lowest group in Model 2, and the likelihood of developing sarcopenia rose in tandem with the increase in IBI. After the ln conversion, its likelihood of developing sarcopenia raised by 42% (OR: 1.42, 95% CI: 1.08–1.87) per unit increase, and the performance was consistent with the results under subgroups. The results were significant at *p* < 0.05 above (Table 2). At the same time, we calculated an *E*‐value of 2.19, which implies that in order to explain the observed OR of 1.42, unmeasured confounders would need to be associated with IBI and sarcopenia with an OR of at least 2.19, which is higher than and far exceeds the OR for measured confounders (Figure S1). Therefore, it seems unlikely that the presence of unmeasured confounders was considered.

**Table 2:** Association between IBI with sarcopenia in the multiple regression model.

Variable	Crude model		Model 1		Model 2	
	OR (95% CI)	*p*	OR (95% CI)	*p*	OR (95% CI)	*p*
IBI	1 (1–1)	0.369	1 (1–1)	0.564	1 (1–1)	0.807
Group						
<4.29	1 (Ref)	—	1 (Ref)	—	1 (Ref)	—
4.29–6.85	1.2 (0.9–1.6)	0.221	1.17 (0.86–1.59)	0.311	1.34 (0.98–1.85)	0.069
6.85–12.34	1.23 (0.93–1.64)	0.152	1.2 (0.88–1.64)	0.248	1.4 (1.05–2.01)	0.025
>12.34	1.61 (1.23–2.12)	0.001	1.63 (1.2–2.21)	0.002	1.94 (1.34–2.81)	0.002
Ln‐IBI	1.17 (1.09–1.27)	<0.001	1.18 (1.08–1.28)	<0.001	1.42 (1.08–1.87)	0.005

*Note:* Crude model: adjusted for none. Model 1: adjusted for sex, age, race, education level, poverty‐to‐income ratio. Model 2: adjusted for Model 1 plus BMI, total serum calcium, HDL, LDL, total cholesterol, alcohol intake, smoking status, hypertension, diabetes, CVD, and CKD.

Abbreviations: CI, confidence interval; OR, odds ratio.

### 3.3. Nonlinear Association Test

**Figure 2:**
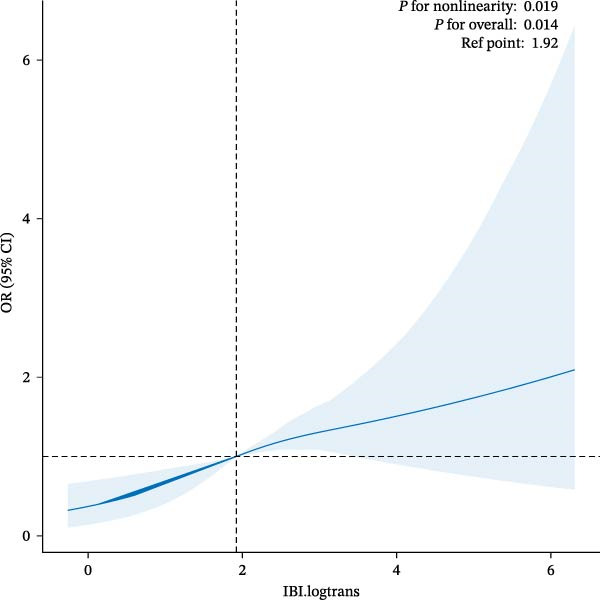
Association between IBI and sarcopenia were evaluated by RCS after adjustment for the covariables.

To explore the nonlinearity of the association between IBI levels and sarcopenia, we used complex weighted RCS curves for testing (Figure 2) (Table 3). After adjusting for covariates, a nonlinear association was found between ln‐transformed IBI levels and sarcopenia (*p* for nonlinearity = 0.019, *p* for overall = 0.014), with an inflection point of 2.37829, before which the risk of suffering from sarcopenia significantly increased as ln‐transformed IBI levels increased and was no longer significant after the inflection point. Disease risk also increased significantly before the inflection point and was no longer significant after the inflection point.

**Table 3:** Analysis of inflection points.

Est.	CI (95%) low	CI (95%) up
2.37829	2.37121	2.38537

### 3.4. Subgroup Analysis

**Figure 3:**
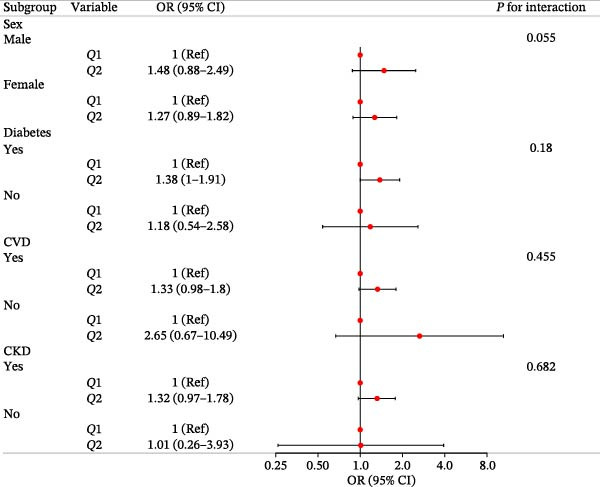
Subgroup analysis and interaction analysis.

We dichotomized IBI for inclusion in the analysis. After adjusting for variables according to Model 2, stratified analyses were conducted based on sex, age, diabetes, CVD, and CKD. In each subgroup, the low IBI group was used as the reference. The high IBI group generally indicated a higher risk of sarcopenia. The absence of an interaction effect was noted, and the results were consistent (Figure 3).

## 4. Discussion

We selected data from NHANES 2015–2018 for this nationally representative cross‐sectional study, and we found consistent results under different treatments for IBI, there was a positive association between IBI levels and sarcopenia and a nonlinear association therein. Our findings remain robust to subgroup and sensitivity analyses.

The causes and mechanisms of sarcopenia are complex and varied, including malnutrition, chronic inflammation, vitamin D deficiency, hypogonadism, physical inactivity, alterations of the neuromuscular junction, intestinal flora imbalance, mitochondrial dysfunction, and reduced muscle protein anabolism [16, 17]. These factors interact with each other to affect the maintenance of muscle mass and muscle function, and chronic low‐grade inflammation is considered to be one of the key pathogenic mechanisms of sarcopenia [4]. As the human organism ages, the immune system becomes more fragile, leading to chronic systemic low‐grade inflammatory responses, accompanied by changes in the levels of related inflammatory cytokines [5, 18]. These inflammatory factors, such as tumor necrosis factor‐alpha (TNF‐α), interleukin‐6 (IL‐6) and CRP have also been identified as factors that play a role in the maintenance of muscle function and mass. CRP exerts its influence on muscle mass through a number of different mechanisms. TNF‐α promotes muscle protein catabolism by activating the p38 MAPK signaling pathway [19]. Research suggests that TNF‐α plays a key role in muscle atrophy and regeneration [20]. TNF‐α may lead to elevated levels of oxidative stress in vivo, damaging muscle cell proteins, lipids, and DNA, and further promoting muscle protein catabolism [21, 22]. IL‐6 inhibits muscle protein synthesis by activating the JAK/STAT pathway [21, 23]. Although IL‐6 stimulates the proliferative capacity of stem cells in the short term, sustained elevation of its serum levels has been associated with muscle atrophy and sarcopenia. Chronic low‐grade inflammation is often accompanied by loss of appetite and inadequate nutrient intake, causing the body to lack the amino acids and other nutrients needed to synthesize muscle proteins, which further inhibits muscle protein synthesis [24]. Other causes of sarcopenia, such as insulin resistance, can be caused by chronic inflammation [25].

The association between chronic inflammation and sarcopenia has been investigated by some researchers. Shi et al. [26] demonstrated that higher inflammation indices are associated with lower muscle mass. Zeng et al. [15] explored an association involving SII and mortality in patients suffering from sarcopenia and, for the first time, a substantial association has been identified between the SII and an elevated probability of mortality in patients diagnosed with sarcopenia. Guo et al. [27] revealed that the CBC‐derived inflammation index, when higher, led to a higher mortality rate of sarcopenia, which is disguised proof of the association between chronic inflammation and sarcopenia. In addition, Benz et al. [28] showed that a higher inflammation index not only increased the mortality rate of sarcopenia but also affected other chronic illnesses. The findings of the present study are in accordance with those of the aforementioned study [28]. Furthermore, skeletal muscle functions as a key metabolic and endocrine organ and plays a vital role in systemic regulation. The loss of muscle mass can further exacerbate inflammatory responses through multiple mechanisms. On one hand, muscle atrophy leads to dysregulated secretion of myokines—such as IL‐6 and myostatin—which directly promote low‐grade systemic inflammation [29]. On the other hand, muscle loss is frequently accompanied by adipose tissue redistribution, especially visceral adipose tissue accumulation. Adipocytes release pro‐inflammatory factors, including leptin and TNF‐α, that further impair muscle function, thereby establishing a vicious cycle between adipose tissue and skeletal muscle [30, 31]. A complex bidirectional relationship exists between sarcopenia and inflammation: while inflammation serves as a core driver of sarcopenia, sarcopenia may in turn amplify inflammatory processes.

In conclusion, our study possesses a number of strengths. Initially, it is imperative to note that the data utilized in this study were meticulously selected from the NHANES database, a renowned source renowned for its accuracy and reliability. Second, we cited the IBI, a new inflammation assessment tool that has many advantages over traditional inflammation indicators. Finally, we found a curvilinear association between IBI and sarcopenia and identified the inflection point, which helps in clinical diagnostic use. Nonetheless, the study is not without its limitations, as outlined below. First, the study used a cross‐sectional design, which limited the inference of causality and failed to clarify the temporal causality between IBI and sarcopenia. Second, no explicit comparisons were made with other indicators of inflammation to explore specific differences in their ability to predict the identification of sarcopenia. And due to the limitations of the questionnaire, many of the subjective tendencies were biased. Finally, the ethnic and geographic specificity of the sample may affect the applicability of the results. Therefore, future studies should conduct large‐scale longitudinal studies to confirm these findings. Although there is still a gap between the current research and clinical application, the potential of IBI as an emerging metric for assessing the risk of sarcopenia and related chronic inflammatory problems remains worthy of further exploration.

## 5. Conclusion

It was demonstrated in the study that there was a significant positive association between IBI levels and sarcopenia, while curve fitting showed that they have a J‐shaped curve association.

**Nomenclature**
IBI: Inflammatory burden index
PIR: Poverty‐to‐income ratio
HDL‐C: High‐density lipoprotein‐cholesterol
LDL‐C: Low‐density lipoprotein‐cholesterol
CVD: Cardiovascular disease
CKD: Chronic kidney disease
OR: Odds ratio
CI: Confidence interval
CDC: Centers for Disease Control and Prevention
NHANES: National Health and Nutrition Examination Survey
Ref: Reference
BMI: Body mass index.

## Author Contributions

**Yunzhe Li and Li Xiao:** conceptualization, methodology, resources, data curation, project administration, writing – original draft, writing – review and editing. **Zixin Luo, Nan Huang, and Maoyuan Wang:** formal analysis, software, investigation, validation, visualization, writing – original draft. **Xiuyun Li, Chenxi Wang, and Shaochun Liu:** data curation, investigation, software, supervision, validation, visualization, writing – review and editing. **Kang Zou**: conceptualization, investigation, project administration, methodology, supervision, writing – review and editing. **Li Xiao**: funding acquisition.

## Funding

This work was supported by the Ganzhou Municipal Health Commission (Grant 2022‐2‐088), the Jiangxi Provincial Administration of Traditional Chinese Medicine (Grant 2022A264), the Health Commission of Jiangxi Province (Grant 202310737), the Ganzhou guiding science and technology plan (Grant GZ2023ZSF131).

## Disclosure

All authors participated in editing, reviewing, and approving the final manuscript.

## Ethics Statement

The requirement for written informed consent was waived for this study, as the data utilized are from the National Health and Nutrition Examination Survey (NHANES), which provides publicly available and de‐identified data with ethical approval already granted by the responsible authorities.

## Consent

Each participant also provided written informed consent.

## Conflicts of Interest

The authors declare no conflicts of interest.

## Supporting Information

Additional supporting information can be found online in the Supporting Information section.

## Supporting information


**Supporting Information** Table S1. Univariate analysis. Figure S1: Curve depicting the range of joint relationships (IBI and Sarcopenia) that may explain away the estimated effect and its confidence interval for the multivariable logistic regression model to predict plaque progression.

## Data Availability

The datasets used and/or analyzed during the current study are available from the NHANES database, https://wwwn.cdc.gov/nchs/nhanes/.
