# Supplementary material for: Elevated Inflammatory Burden Index Is Association With Increased Sarcopenia: A Population‐Based Study
Source: Mediators Inflamm. 2026 Mar 26;2026:9991220. doi: 10.1155/mi/9991220 (PMC13140181; doi:10.1155/mi/9991220)
Supplement: Supplementary file 1 — Supporting Information Table S1. Univariate analysis. Figure S1: Curve depicting the range of joint relationships (IBI and Sarcopenia) that may explain away the estimated effect and its confidence interval for the multivariable logistic regression model to predict plaque progression. [file MI-2026-9991220-s001.docx]

**TABLE S1** Univariate analysis.

| Variable | OR_95CI | *P*_value |
| --- | --- | --- |
| Sex, n (%) |  |  |
| Male | Ref |  |
| Female | 0.91 (0.75~1.1) | 0.339 |
| Age(year) | 1.04 (1.03~1.05) | <0.001 |
| Race, n (%) |  |  |
| Mexican American | Ref |  |
| Other Hispanic | 0.54 (0.4~0.72) | <0.001 |
| Non-Hispanic White | 0.25 (0.19~0.32) | <0.001 |
| Non-Hispanic Black | 0.08 (0.05~0.12) | <0.001 |
| Other Race | 0.33 (0.25~0.44) | <0.001 |
| Education level, n (%) |  |  |
| Non-graduate | Ref |  |
| high school graduation | 0.4 (0.33~0.49) | <0.001 |
| PIR, n (%) |  |  |
| ≤1 | Ref |  |
| >1 | 0.57 (0.46~0.71) | <0.001 |
| BMI, Mean ± SD | 1.12 (1.1~1.13) | <0.001 |
| Total Calcium, mean ± SD | 0.47 (0.36~0.63) | <0.001 |
| HDL-C, mean ± SD | 0.98 (0.97~0.98) | <0.001 |
| LDL-C, mean ± SD | 1 (1~1.01) | 0.006 |
| Total Cholesterol, mean ± SD | 1 (1~1.01) | <0.001 |
| Alcohol intake, n (%) |  |  |
| Current | Ref |  |
| Former | 1.24 (1~1.54) | 0.048 |
| Never | 2.07 (1.58~2.72) | <0.001 |
| Smoking status, n (%) |  |  |
| Current | Ref |  |
| Former | 1.19 (0.91~1.57) | 0.204 |
| Never | 1.46 (1.18~1.8) | <0.001 |
| Hypertension, n (%) |  |  |
| No | Ref |  |
| Yes | 0.76 (0.61~0.94) | 0.011 |
| Diabetes, n (%) |  |  |
| No | Ref |  |
| Yes | 2.67 (2.03~3.52) | <0.001 |
| CVD, n (%) |  |  |
| No | Ref |  |
| Yes | 1.93 (1.29~2.88) | 0.001 |
| CKD, n (%) |  |  |
| No | Ref |  |
| Yes | 1.81 (1.37~2.41) | <0.001 |

**
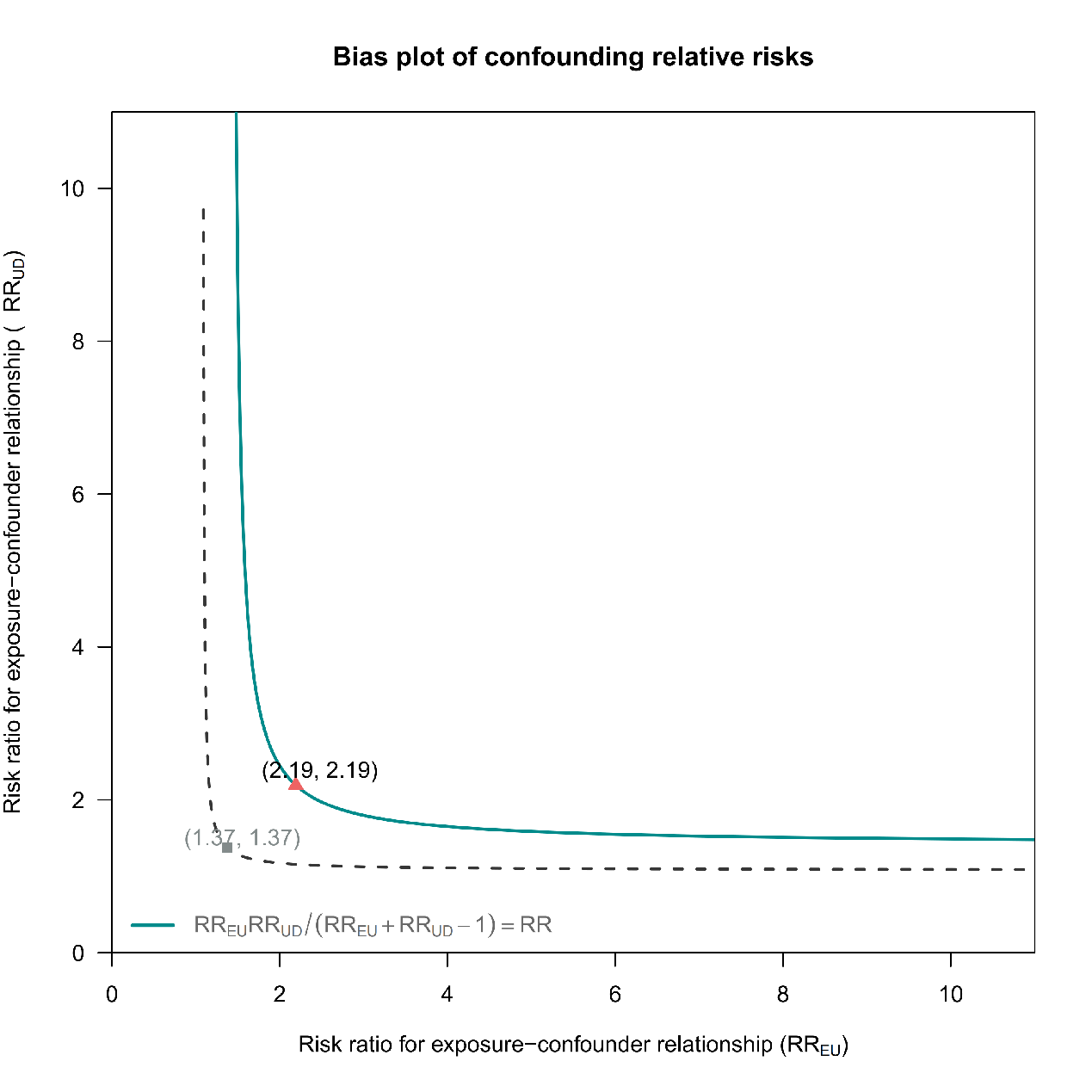
**

**FIGURE S1** Curve depicting the range of joint relationships (IBI and Sarcopenia) that may explain away the estimated effect and its confidence interval for the multivariable logistic regression model to predict plaque progression.
